# Selective copper(II) acetate and potassium iodide catalyzed oxidation of aminals to dihydroquinazoline and quinazolinone alkaloids

**DOI:** 10.3762/bjoc.9.135

**Published:** 2013-06-20

**Authors:** Matthew T Richers, Chenfei Zhao, Daniel Seidel

**Affiliations:** 1 Department of Chemistry and Chemical Biology, Rutgers, The State University of New Jersey, Piscataway, New Jersey 08854, USA

**Keywords:** aminal, copper, oxygen, *tert*-butylhydroperoxide, quinazoline alkaloid

## Abstract

Copper(II) acetate/acetic acid/O_2_ and potassium iodide/*tert*-butylhydroperoxide systems are shown to affect the selective oxidation of ring-fused aminals to dihydroquinazolines and quinazolinones, respectively. These methods enable the facile preparation of a number of quinazoline alkaloid natural products and their analogues.

## Introduction

**Figure 1:**
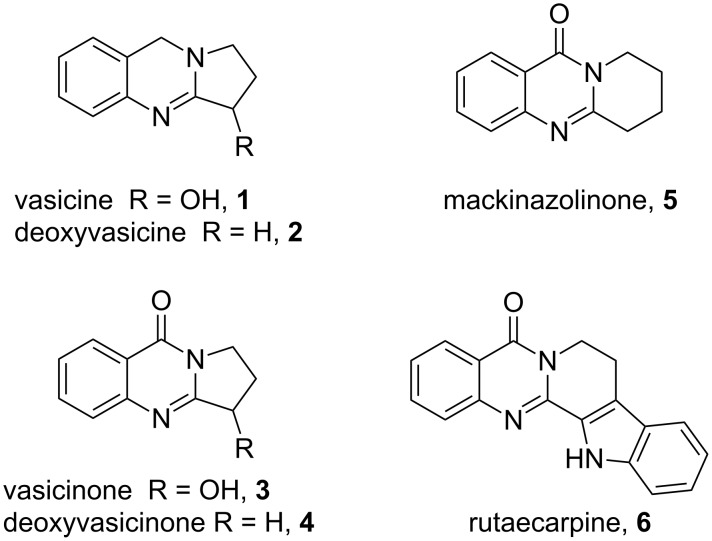
Examples of naturally occurring quinazoline alkaloids.

Quinazoline alkaloids are a class of naturally occurring compounds with a range of medicinal properties and have been indicated for use as bronchodilators, vasodilators, anti-inflammatory agents and acetylcholinesterase inhibitors [1–5]. Many of the plants these products have been isolated from, such as *Adhatoda vasica, Peganum harmala* and *Evodia rutaecarpa*, have been used in folk medicine for centuries [6–9]. Since the original isolation of vasicine (**1**, Figure 1) in 1888 [10], the biological properties of this class of alkaloids have been extensively studied.

**Figure 2:**
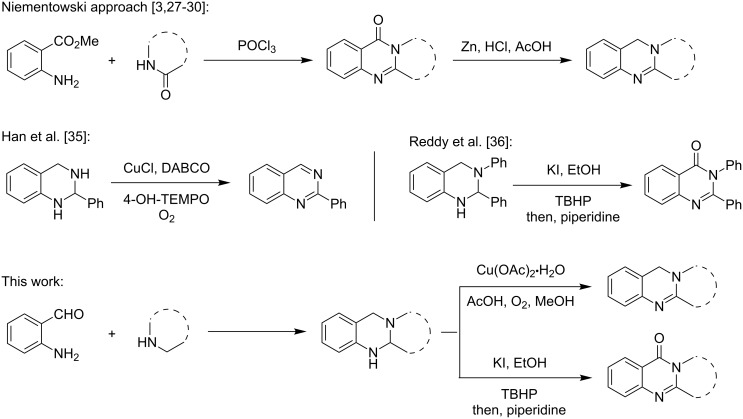
Different approaches to the synthesis of quinazoline alkaloid structures.

A number of synthetic strategies have been employed to gain access to quinazoline alkaloids [5,11–26]. Perhaps the most common method involves the condensation of an *ortho*-aminobenzoic ester with a lactam promoted by phosphoryl chloride, known as the Niementowski reaction [3,27–30] (Figure 2). The availability, or lack thereof, of the corresponding lactam can determine the length and efficiency of the route. Access to the sometimes more biologically active dihydroquinazolines, such as deoxyvasicine (**2**), from quinazolinones requires a subsequent reduction of the amide. In 2008, our group reported the syntheses of deoxyvasicinone (**4**) and rutaecarpine (**6**) by the potassium permanganate promoted oxidation of aminals, which in turn were obtained from the condensation of *ortho*-aminobenzaldehydes and simple secondary amines [31–32]. A number of these aminal precursors were prepared in generally good to excellent yields with the scope encompassing various cyclic amines and substituents on the aminobenzaldehyde aryl ring. Since then, we have demonstrated that the reaction can be run on a multigram scale [33] and have shown that dihydroquinazolines vasicine (**1**) and deoxyvasicine (**2**) can be synthesized from their corresponding aminals by using an iodine-promoted oxidation [34]. While resulting in good yields, these oxidations have the drawback of requiring large amounts of a strong oxidant for the permanganate oxidation and the necessity of stoichiometric *n*-butyllithium for the iodine reaction.

The conversion of the aminals formed from the condensation of aminobenzaldehydes and secondary amines to the corresponding dihydroquinazoline and quinazolinone structures under mild and catalytic conditions would be preferable to using harsh oxidants and strong bases. Han et al. have recently shown the ability of copper salts, in conjunction with oxygen, to catalyze oxidations of 2-substituted tetrahydroquinazoline aminals to quinazolines [35] (Figure 2). In addition, Reddy and co-workers have developed a catalytic system in which 2,3-substituted tetrahydroquinazoline aminals are converted to quinazolinones using *tert-*butylhydroperoxide (TBHP) and catalytic potassium iodide [36–37]. While these examples deal with the oxidation of bicyclic aminals, we were interested in developing methods to create dihydroquinazoline and quinazolinone alkaloids from ring-fused aminals. Here we present catalytic methods for the synthesis of both these compound classes from aminals using Cu(OAc)_2_/O_2_/AcOH and KI/TBHP systems, respectively.

## Results and Discussion

### Copper-catalyzed oxidations of aminals to dihydroquinazolines

Copper-catalyzed oxidation reactions have received a great deal of interest in recent years [38–44]. Han’s copper-catalyzed method for the synthesis of aminals to quinazolines results in high yields [35], but the process is not applicable to mono-oxidation as dihydroquinazolines are not isolated as products in these reactions. We set out to develop a method for the synthesis of dihydroquinazolines that would prevent further oxidation at the benzylic position. A factor complicating this effort was that dihydroquinazolines like deoxyvasicine (**2**) are known to auto-oxidize to their quinazolinone counterparts by exposure to air [3,45–47]. We initiated our efforts by exposing aminal **7** to stoichiometric amounts of CuCl_2_ in acetonitrile under a nitrogen atmosphere, which led to the formation of **2** in 81% yield (Table 1, entry 1). To improve the efficiency of the process, catalytic conditions were subsequently evaluated. When aminal **7** was heated under reflux in an oxygen atmosphere and in the presence of 20 mol % of CuCl_2_, **2** was only observed in trace amounts; deoxyvasicinone (**4**) and peroxide **8** were also formed as products. Switching the catalyst to Cu(OAc)_2_ led to a 15% yield of the desired product **2**, but the process was still unselective.

**Table 1:** Optimization of conditions for deoxyvasicine (**2**) formation.^a^

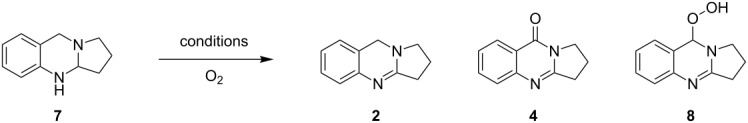								

Entry	Solvent (0.2 M)	Catalyst (mol %)	Acid (equiv)	Temp. (°C)	Time (h)	Yield of **2** (%)	Yield of **4** (%)	Yield of **8** (%)

1^b^	MeCN	CuCl_2_∙2H_2_O (100)	–	rt	6	81	–	–
2	MeCN	CuCl_2_∙2H_2_O (20)	–	81	2	trace	14	10
3	MeCN	Cu(OAc)_2_∙H_2_O (20)	–	81	3	15	17	trace
4	MeCN	Cu(OAc)_2_∙H_2_O (20)	AcOH (1.1)	81	3	53	–	–
5	MeOH	Cu(OAc)_2_∙H_2_O (20)	AcOH (1.1)	65	4	81	–	–
6	MeOH	Cu(OAc)_2_∙H_2_O (20)	–	65	4	33	6	24
7	AcOH	Cu(OAc)_2_∙H_2_O (20)	–	80	24	18^c^	–	–
8	DMF	Cu(OAc)_2_∙H_2_O (20)	AcOH (1.1)	80	4	17	20	–
9	MeOH	Cu(2-EH)_2_ (20)	2-EHA (1.1)	65	12	71	–	–
10	MeOH	CuBr (20)	AcOH (1.1)	65	8	72	–	–
11	EtOH^d^	Cu(OAc)_2_∙H_2_O (20)	AcOH (1.1)	78	1.5	73	–	trace
12	MeOH	Cu(OAc)_2_∙H_2_O (10)	AcOH (1.1)	65	18	67	–	trace
13	MeOH	Cu(OAc)_2_∙H_2_O (20)	AcOH (1.1)	40	24	61	trace	trace
14	MeOH	Cu(acac)_2_ (10)	AcOH (1.1)	65	24	68^c^	trace	trace

^a^Reactions were performed on a 0.25 mmol scale. Cu(2-EH)_2_ = copper(II) 2-ethylhexanoate. 2-EHA = 2-ethylhexanoic acid. ^b^Nitrogen atmosphere. ^c^The reaction was incomplete. ^d^95% Solution.

It appears that the first oxidation occurs exclusively at the aminal site to form deoxyvasicine (**2**). The presence of the amidine moiety apparently activates the molecule for oxidation at the benzylic position; we have observed that samples of aminal **7** can remain stable in the freezer for years, whereas **2** begins to convert to **4** within a day when exposed to atmospheric oxygen. Considering this, we reasoned that addition of a weak acid to protonate the relatively basic amidine moiety of **2** might deactivate the benzylic position toward oxidation while not interfering with the initial aminal oxidation. Indeed, using 1.1 equivalents of acetic acid as an additive with catalytic Cu(OAc)_2_ in acetonitrile led to the formation of **2** in 53% yield without formation of **4** and **8** (Table 1, entry 4). A simple change of the solvent from acetonitrile to methanol drastically improved the yield of **2** to 81% (Table 1, entry 5). A number of different copper salts, solvents and acids were then evaluated, but none of the changes led to a further improvement in yield. It appears that under certain conditions catalyst deactivation via copper oxide formation decreased the catalyst turnover and consequently product yields.

Using the optimized reaction conditions, a range of different aminals were selectively oxidized to the corresponding dihydroquinazolines (Table 2). In general, these products were obtained in moderate to good yields. Product **10**, containing a piperidine ring, required a higher reaction temperature and resulted in a lower yield than the corresponding pyrrolidine and azepane products (**2** and **12**, respectively). While differences in conformation may in part account for the observed differences in reactivity (X-ray crystal structures of aminals containing pyrrolidine and piperidine revealed that the pyrrolidine-containing aminal adopts a bent structure, whereas the piperidine aminal appears relatively strain-free [34]), this finding likely relates to the reduced propensity of six-membered rings to engage in reactions that form exocyclic double bonds. The isolation of azepinoquinazoline **12** in 73% yield was gratifying but somewhat unexpected since Decker reported that samples of the compound completely oxidized to quinazolinone **23** when exposed to air for 24 h [3]. This demonstrates the need for acetic acid to protonate the amidine, preventing further oxidation. While product **16** was obtained in good yields from tetrahydroisoquinoline-aminal **15**, rutaecarpane-derived product **18** was formed in only 47% yield, apparently due to unidentified side-reactions. The reaction leading to the synthesis of the dibromo- analogue of deoxyvasicine **20**, even under elevated temperature and extended reaction time, still did not reach completion after 3 days. The attenuated reactivity of aminal **19** is most likely the result of the decreased electron density on the anilinic nitrogen.

**Table 2:** Scope of the copper-catalyzed conversion of aminals to dihydroquinazolines.^a^

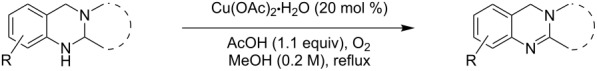				

Entry	Starting material	Product	Time (h)	Yield (%)

1	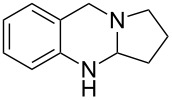 **7**	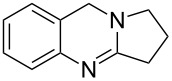 **2**	7	86
2^b,c^	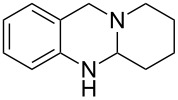 **9**	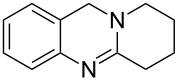 **10**	8	57
3^c^	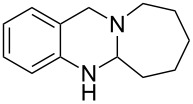 **11**	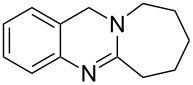 **12**	7	73
4	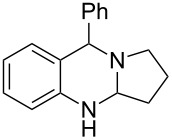 **13**	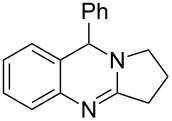 **14**	24	72
5	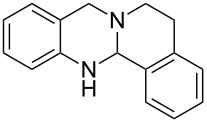 **15**	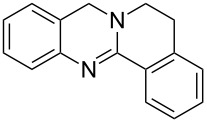 **16**	8	82
6	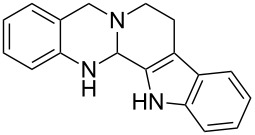 **17**	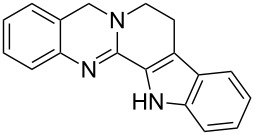 **18**	4	47
7^b,d^	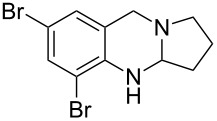 **19**	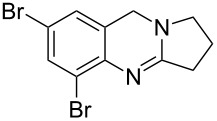 **20**	72	18

^a^Reactions run on a 1 mmol scale. ^b^EtOH used as solvent. ^c^0.5 mmol scale. ^d^Reaction incomplete.

### KI-catalyzed oxidations of aminals to quinazolinones

Different conditions for the direct catalytic oxidation of aminals to quinazolinones were also explored. The use of Cu(OAc)_2_ and methanol, while appropriate for furnishing deoxyvasicine (**2**) from aminal **7**, did not result in satisfactory yields of deoxyvasicinone (**4**, Table 1). Attempts to use other copper(I) or copper(II) salts and solvents under oxygen without the addition of acid to promote the full oxidation of aminal **21** to deoxyvasicinone (**4**) were met with disappointment, with yields of **4** for these conditions reaching a maximum of around 40% (Table 3). In most cases, peroxide **8** was observed as a major side product. The Cu/TEMPO/DABCO catalyst system employed by Han et al. [35] for the oxidation of aminals to quinazolines provided an increased yield of 50% (Table 3, entry 9). The best yields were obtained by using the conditions developed by Reddy and co-workers [36], namely the combined use of catalytic amounts of potassium iodide (20 mol %) and excess TBHP (5 equiv), followed by the addition of piperidine. In this instance, deoxyvasicinone was isolated in 80% yield (Table 3, entry 12). In the course of this reaction, the TBHP adduct **22** is formed as an intermediate that is subsequently converted to the quinazolinone upon addition of piperidine. A slight modification of Reddy’s conditions, in which piperidine was added directly to the solution after 36 hours instead of the removal of solvent from the intermediate peroxide beforehand, resulted in identical yields.

**Table 3:** Optimization of conditions for deoxyvasicinone (**4**) formation.^a^

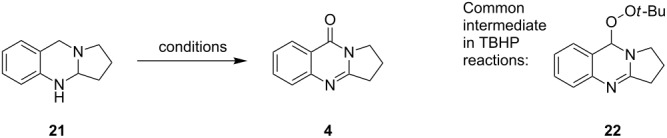							

Entry	Solvent (0.2 M)	Catalyst (mol %)	Oxidant (equiv)	Additive (equiv)	Temp. (°C)	Time (h)	Yield of **4** (%)

1	DMSO	CuBr (20)	O_2_	–	100	2	21
2	DMSO	CuBr (20)	O_2_	DBU (0.4)	100	17	25
3	DMSO	CuBr (20)	O_2_	DBU (2)	100	3	22
4	DMSO	CuBr (20)	O_2_	–	60	3	28
5	MeCN	CuBr(10)	O_2_	–	80	24	43
6	DMF	CuBr(10)	O_2_	–	80	24	42
7	DMSO	CuI (20)	O_2_	–	60	3	29
8	MeCN	CuCl_2_·2H_2_O (20)	O_2_	–	50	5	19
9	MeCN	CuCl (10)	O_2_	DABCO (0.1), TEMPO (0.05)	80	12	50
10	DMSO	CuCl (10)	O_2_	DABCO (0.1), TEMPO (0.05)	100	3	38
11	PhMe	CuBr (20)	TBHP (5)	piperidine^b^ (5)	rt	0.5	61
12	EtOH	KI (20)	TBHP (5)	piperidine^b^ (5)	rt	36	80

^a^Reactions run on a 0.25 mmol scale. DBU = 1,8-Diazabicyclo[5.4.0]undec-7-ene. DABCO = 1,4-Diazabicyclo[2.2.2]octane. TEMPO = 2,2,6,6-Tetramethylpiperidine-1-oxy radical. ^b^Piperidine was added at the end of the reaction and the reaction mixture was heated at 50 °C for 1 h.

Using the optimized conditions, a range of different quinazolinones were synthesized (Table 4). In general, yields were moderate to good for substrates with varying ring sizes. In this manner the natural products deoxyvasicine (**4**), mackinazolinone (**5**) and rutaecarpine (**6**) were prepared, in addition to the azepinoquinazolone **23**, which has been demonstrated to be a more effective antitussive agent than codeine [48]. Dibromo-deoxyvasicinone analogue **25** was obtained in relatively high yield (88%) whereas the corresponding analogue of mackinazolinone (**27**) was obtained in only 50% yield.

**Table 4:** Scope of KI-catalyzed conversion of aminals to quinazolinones.^a^

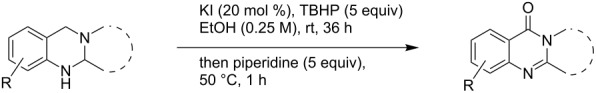			

Entry	Starting material	Product	Yield [%]

1	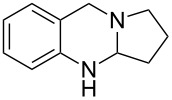 **7**	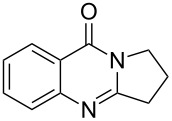 **4**	84
2^b^	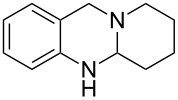 **9**	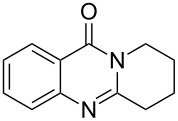 **5**	59
3^b^	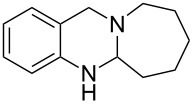 **11**	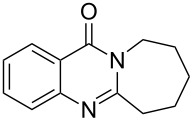 **23**	69
4	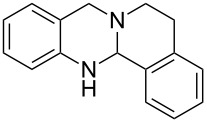 **15**	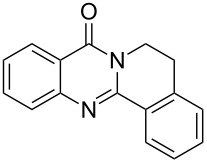 **24**	60
5	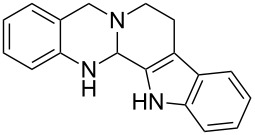 **17**	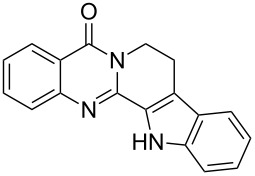 **6**	58
6	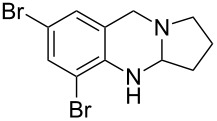 **19**	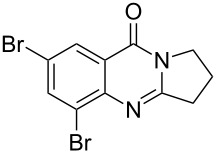 **25**	88
7	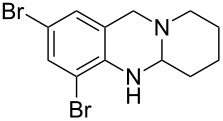 **26**	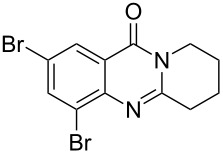 **27**	50

^a^Reactions run on a 1 mmol scale. ^b^0.5 mmol scale.

**Scheme 1:**
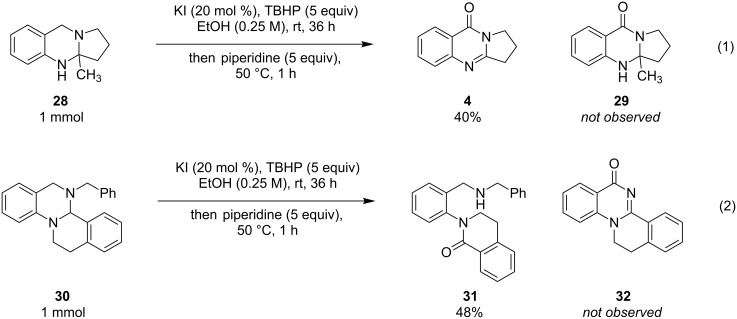
Oxidation of other aminal systems.

Interestingly, when quaternary aminal **28** was subjected to oxidative conditions in an attempt to prepare compound **29**, deoxyvasicinone (**4**) was obtained as the major product in a process that involved demethylation (Scheme 1, reaction 1). The demethylation of aminals has been previously reported in cases where the product achieves aromaticity [49–51], which is presumably the driving force for this transformation. Aminal **30**, which contains two tertiary amines and is readily obtainable by an acid-promoted hydride shift process [52–54], was also exposed to oxidative conditions (Scheme 1, reaction 2). We had hypothesized that quinazolinone **32** might be formed in this reaction by the debenzylation of an intermediate iminium ion. However, the major product from this reaction was identified to be **31**, the apparent product of iminium hydrolysis.

## Conclusion

We have demonstrated that quinazoline alkaloids and their analogues can be synthesized from aminals by using Cu(OAc)_2_/O_2_/AcOH and KI/TBHP catalyst systems. The use of acetic acid in addition to oxygen and catalytic copper(II) salts was determined to prevent overoxidation of dihydroquinazolines, allowing access to these structures under mild conditions. A number of natural products and their analogues were obtainable by these methods, which should facilitate the preparation of novel materials for biological studies.

## Supporting Information

File 1 Experimental details, characterization data and ^1^H and ^13^C NMR spectra for all new compounds.

## References

[1] D’yakonov A L, Telezhenetskaya M V (1997). Chem Nat Compd.

[2] Sheu J-R (1999). Cardiovasc Drug Rev.

[3] Decker M (2005). Eur J Med Chem.

[4] Rachana S, Ujata B, Mamta P, Priyanka K M, Sonam S (2011). Indo Global J Pharm Sci.

[5] Nepali K, Sharma S, Ojha R, Dhar K L (2013). Med Chem Res.

[6] Jia S, Hu C (2010). Molecules.

[7] Dhankhar S, Kaur R, Ruhil S, Balhara M, Dhankhar S, Chhillar A K (2011). Afr J Plant Sci.

[8] Corrêa G M, Alcântara A F de C (2012). Rev Bras Farmacogn.

[9] Asgarpanah J, Ramezanloo F (2012). Afr J Pharm Pharmacol.

[10] Hooper D (1888). Pharm J Trans.

[11] Bergman J, Bergman S (1981). Heterocycles.

[12] Mhaske S B, Argade N P (2001). J Org Chem.

[13] Yadav J S, Reddy B V S (2002). Tetrahedron Lett.

[14] Mhaske S B, Argade N P (2006). Tetrahedron.

[15] Bowman W R, Elsegood M R J, Stein T, Weaver G W (2007). Org Biomol Chem.

[16] Zhou J, Fu L, Lv M, Liu J, Pei D, Ding K (2008). Synthesis.

[17] Kraus G A, Guo H (2009). J Org Chem.

[18] Giri R, Lam J K, Yu J-Q (2010). J Am Chem Soc.

[19] Kshirsagar U A, Puranik V G, Argade N P (2010). J Org Chem.

[20] Dabiri M, Salehi P, Bahramnejad M, Alizadeh M (2010). Monatsh Chem.

[21] Zeng F, Alper H (2010). Org Lett.

[22] Kshirsagar U A, Argade N P (2010). Org Lett.

[23] Tseng M-C, Cheng H-T, Shen M-J, Chu Y-H (2011). Org Lett.

[24] Granger B A, Kaneda K, Martin S F (2011). Org Lett.

[25] Fang J, Zhou J (2012). Org Biomol Chem.

[26] Murai K, Komatsu H, Nagao R, Fujioka H (2012). Org Lett.

[27] von Niementowski S (1895). J Prakt Chem.

[28] Pachter I, Suld G (1960). J Org Chem.

[29] D’yakonov A L, Telezhenetskaya M V, Yunusov L Yu (1986). Chem Nat Compd.

[30] Lee E S, Park J-G, Jahng Y (2003). Tetrahedron Lett.

[31] Zhang C, De C K, Mal R, Seidel D (2008). J Am Chem Soc.

[32] Dieckmann A, Richers M T, Platonova A Yu, Zhang C, Seidel D, Houk K N (2013). J Org Chem.

[33] Zhang C, De C K, Seidel D (2012). Org Synth.

[34] Richers M T, Deb I, Platonova A Y, Zhang C, Seidel D (2013). Synthesis.

[35] Han B, Yang X-L, Wang C, Bai Y-W, Pan T-C, Chen X, Yu W (2012). J Org Chem.

[36] Kumar R A, Maheswari C U, Ghantasala S, Jyothi C, Reddy K R (2011). Adv Synth Catal.

[37] Kumar R A, Saidulu G, Prasad K R, Kumar G S, Sridhar B, Reddy K R (2012). Adv Synth Catal.

[38] Li C-J (2009). Acc Chem Res.

[39] Punniyamurthy T, Rout L (2008). Coord Chem Rev.

[40] Wendlandt A E, Suess A M, Stahl S S (2011). Angew Chem, Int Ed.

[41] Hirano K, Miura M (2012). Chem Commun.

[42] Zhang C, Tang C, Jiao N (2012). Chem Soc Rev.

[43] Jones K M, Klussmann M (2012). Synlett.

[44] Ratnikov M O, Doyle M P (2013). J Am Chem Soc.

[45] Mehta D R, Naravane J S, Desai R M (1963). J Org Chem.

[46] Johns S R, Lamberton J A, Suares H (1985). Aust J Chem.

[47] Nakagawa Y, Stevens R V (1988). J Org Chem.

[48] Nepali K, Bande M S, Sapra S, Garg A, Kumar S, Sharma P, Goyal R, Satti N K, Suri O P, Dhar K L (2012). Med Chem Res.

[49] Elderfield R C, Kreysa F J, Dunn J H, Humphreys D D (1947). J Am Chem Soc.

[50] Yamazaki C (1981). J Org Chem.

[51] Schulze K, Richter C, Ludwig R (1989). Tetrahedron Lett.

[52] Zhang C, Murarka S, Seidel D (2009). J Org Chem.

[53] Mori K, Ohshima Y, Ehara K, Akiyama T (2009). Chem Lett.

[54] He Y-P, Du Y-L, Luo S-W, Gong L-Z (2011). Tetrahedron Lett.

