# Supplementary material for: Selective copper(II) acetate and potassium iodide catalyzed oxidation of aminals to dihydroquinazoline and quinazolinone alkaloids
Source: Beilstein J Org Chem. 2013 Jun 20;9:1194–201. doi: 10.3762/bjoc.9.135 (PMC3701376; doi:10.3762/bjoc.9.135)

Supporting Information
for
Selective copper(II) acetate and potassium iodide catalyzed oxidation of aminals to dihydroquinazoline and quinazolinone alkaloids

Matthew T. Richers, Chenfei Zhao and Daniel Seidel*

Address: [1]Department of Chemistry and Chemical Biology, Rutgers, The State University of New Jersey, Piscataway, New Jersey 08854, USA

Email: Daniel Seidel - seidel@rutchem.rutgers.edu

* Corresponding author

Experimental details, characterization data and ^{1}H and ^{13}C NMR spectra for all new compounds

General information: Reagents and solvents were purchased from commercial sources and were used as received unless otherwise stated. $Cu(OAc)_2 \cdot H_2O$ was purchased from Fischer Scientific and was used as received. HPLC grade methanol was purchased from Sigma Aldrich and was used as received. Glacial acetic acid was purchased from EMD and was used as received. 5.5 M *tert*-butylhydroperoxide solution in decane was purchased from Fluka and was titrated according to the literature [1]. Potassium iodide was purchased from Mallinckrodt and was used as received. Ethanol (absolute) was purchased from Pharmco-Aaper and used as received. Piperidine and azepane were purchased from Sigma Aldrich and were distilled prior to use. Microwave reactions were carried out in a CEM Discover reactor. Silicon carbide (SiC) passive heating elements were purchased from Anton Paar. Purification of reaction products was carried out by flash column chromatography using Sorbent Technologies Standard Grade silica gel (60 Å, 230–400 mesh). Analytical thin-layer chromatography was performed on EM Reagent 0.25 mm silica gel 60 F_{254} plates. Visualization was accomplished with UV light, potassium permanganate, and Dragendorff-Munier stains followed by heating. Melting points were recorded on a Thomas Hoover capillary melting point apparatus and are uncorrected. Infrared spectra were recorded on an ATI Mattson Genesis Series FT-Infrared spectrophotometer. Proton nuclear magnetic resonance spectra (^{1}H NMR) were recorded on a Varian VNMRS-500 MHz and are reported in ppm using the solvent as an the internal standard ($CDCl_3$ at 7.26 ppm). Data are reported as app = apparent, s = singlet, d = doublet, t = triplet, q = quartet, p = pentet, m =

multiplet, comp = complex, br = broad; and coupling constant(s) in Hz. Proton-decoupled carbon nuclear magnetic resonance spectra (^{13}C NMR) were recorded on a Varian VNMRS-500 MHz and are reported in ppm using the solvent as an internal standard ($CDCl_3$ at 77.0 ppm). Mass spectra were recorded on a Finnigan LCQ-DUO mass spectrometer. Aminals **7** [2], **9** [3], **13** [3], **15** [2], **17** [2], **19** [2], **26** [2], **28** [4] and **30** [5] were prepared according to the literature. Products **2** [6], **4** [2], **5** [7], **6** [2], **16** [8], **18** [9], **23** [10], **24** [11], and **25** [12], were previously reported and their published characterization data matched our own in all regards.

General copper-oxidation procedure: A round bottom flask was charged with a magnetic stir bar, aminal (1 mmol), MeOH (5 mL, 0.2 M), acetic acid (1.1 equiv) and $Cu(OAc)_2{\cdot}H_2O$ (0.2 equiv). A septum was placed over the flask and the content of an oxygen-filled balloon was flushed through the flask. A second balloon was placed on top of a reflux condenser, which was placed on top of the flask, and the mixture was heated under reflux until the starting material was consumed as determined by TLC. Solvent was then removed in vacuo and the residue was loaded onto a column in celite and purified by silica gel chromatography. The product was then subjected to silica gel chromatography a second time to remove residual colored impurities.

General TBHP-oxidation procedure: To a round bottom flask charged with a magnetic stir bar, aminal (1 mmol), absolute EtOH (4 mL, 0.25 M), and KI (0.2 equiv) was added *tert*-butylhydroperoxide solution in decane (5.4 M, 5 equiv) dropwise. The mixture was stirred under nitrogen at room temperature for 36 h. After this time, piperidine (5 equiv) was added, and the solution was heated at 50 °C for 1 h, followed by cooling to room temperature. The solvent was removed in vacuo and the product was purified by silica gel chromatography.

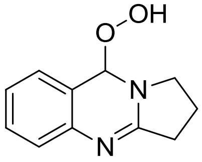

Compound 8: Following the general copper-oxidation procedure but without addition of acetic acid, **8** was obtained from aminal **7** (0.25 mmol scale) after 4 h. **8** was isolated as a tan solid in 24% yield, in addition to **2** (33%) and **4** (6%). Characterization data for **8**: R_f 0.35 in EtOAc/MeOH 80:20 v/v; ^{1}H NMR (500 MHz, $CDCl_3$) δ 7.41 (app d, J = 7.6 Hz, 1H), 7.29 (app t, J = 7.4 Hz, 1H), 7.14 (app t, J = 7.4 Hz, 1H), 7.10 (app d, J = 7.9 Hz, 1H), 5.89 (s, 1H), 3.99 (app q, J = 8.7 Hz, 1H), 3.39 (app td, J = 9.7, 3.1, 1H), 2.08 (ddd, J = 17.0, 9.4, 3.4 Hz, 1H), 1.83–1.58 (comp, 2H), 0.90 (app dt, J = 16.5, 9.7, Hz, 1H); ^{13}C NMR (125 MHz, $CDCl_3$) δ 162.3, 142.2, 128.7, 128.6, 124.1, 123.2, 122.9, 76.6, 48.3, 30.0, 18.4; ESIMS (*m/z*): 203.2 $[M - H]^+$, 171.3 $[M - O_2H]^+$.

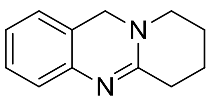

Compound 10: Following the general copper-oxidation procedure but using absolute ethanol instead of methanol, **10** was obtained from aminal **9** (0.5 mmol scale) after 8 h. **10** was isolated as a tan solid in 57% yield (0.0534 g). R_f 0.10 in EtOAc/MeOH/Et_3N 89:10:1 v/v/v); mp 89–92 ºC; IR (KBr) 3041, 2945, 2830, 2360, 2343, 1587, 1560, 1496, 1319, 1160, 1095, 1027, 779, 764, 722, 681 cm^{-1}; ^{1}H NMR (500 MHz, $CDCl_3$) 7.12 (app t, J = 7.6 Hz, 1H), 7.01 (app d, J = 8.0 Hz, 1H), 6.92 (app td, J = 7.4, 1.3 Hz, 1H), 6.83 (app d, J = 7.4 Hz, 1H), 4.42 (s, 2H), 3.07 (app t, J = 6.1 Hz, 2H), 2.53 (app t, J = 6.5 Hz, 2H), 1.92–1.80 (comp, 2H), 1.80–1.69 (comp, 2H); ^{13}C NMR (125 MHz, $CDCl_3$) δ 156.7, 142.1, 128.2, 124.8, 123.6, 122.8, 120.2, 51.1, 50.1, 31.5, 23.2, 20.4; ESIMS (*m/z*): 187.2 $[M + H]^+$.

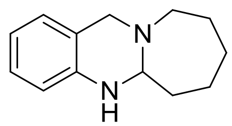

Compound 11: A 10 mL microwave reaction tube was charged with a 10 x 8 mm SiC passive heating element, 2-aminobenzaldehyde [13] (0.061 g, 0.5 mmol), *n*-BuOH (2 mL) and azepane (1.5 mL, 2.5 equiv). The reaction tube was sealed with a Teflon-lined snap cap, and heated in a microwave reactor at 225 °C for 1 h (***Note: SiC passive heating elements must not be used in conjunction with stir bars because they may score glass and cause vessel failure***). After cooling with compressed air flow, the reaction mixture was transferred to a round bottom flask and the vessel was rinsed with EtOAc (4 x 2 mL). Solvent was then removed *in vacuo* and the residue was loaded onto a short column and purified by silica gel chromatography. **11** was isolated as a yellow oil in 69% yield (0.0699 g). R_f 0.19 in EtOAc/MeOH 90:10 v/v); IR (KBr) 3392, 3048, 2927, 2850, 2754, 1608, 1496, 1358, 1283, 1258, 1145, 1069, 1003, 971, 746 cm^{-1}; ^1H NMR (500 MHz, $CDCl_3$) 7.00 (app t, *J* = 8.0 Hz, 1H), 6.89 (app d, *J* = 7.4 Hz, 1H), 6.65 (app td, *J* = 7.4, 1.2 Hz, 1H), 6.53 (dd, *J* = 8.0, 1.1 Hz, 1H), 4.15–4.04 (comp, 2H), 3.75 (d, *J* = 15.1 Hz, 1H), 2.89 (ddd, *J* = 13.9, 6.0, 4.6 Hz, 1H), 2.56 (ddd, *J* = 14.7, 6.0, 4.6 Hz, 1H), 2.04–1.94 (m, 1H), 1.87–1.71 (comp, 3H), 1.71–1.55 (comp, 4H); ^{13}C NMR (125 MHz, $CDCl_3$) δ 143.4, 127.1, 126.7, 120.8, 117.8, 114.6, 72.3, 57.8, 50.8, 35.4, 28.9, 28.8, 21.9; ESIMS (*m/z*): 203.1 $[M + H]^+$.

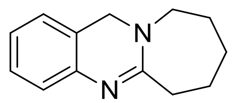

Compound 12: Following the general copper-oxidation procedure, **12** was obtained from aminal **11** (0.5 mmol scale) after 7 h. **12** was isolated as a tan solid in 73% yield (0.0730 g). R_f 0.13 in EtOAc/MeOH/Et_3N 89:10:1 v/v/v); mp: 80–83 ºC; IR (KBr) 2923, 2854, 1586, 1563, 1493, 1455, 1441, 1342, 1274, 1194, 1142, 1085, 981, 765 cm^{-1}; ^1H NMR (500 MHz, $CDCl_3$) 7.13 (app td, *J* = 7.7, 1.5 Hz, 1H), 7.02 (dd, *J* = 7.8, 1.2 Hz, 1H), 6.94 (app td, *J* = 7.4, 1.3 Hz, 1H), 6.82 (app d, *J* = 7.4 Hz, 1H), 4.59 (s, 2H), 3.39–3.25 (comp, 2H), 2.65–2.51 (comp, 2H), 1.86–1.60 (comp, 6H); ^{13}C NMR (125 MHz, $CDCl_3$) δ 163.0, 142.7, 128.2, 124.8, 123.9, 123.3, 121.2, 52.6, 51.5, 36.6, 29.8, 26.9, 25.3; ESIMS (*m/z*): 201.3 $[M + H]^+$.

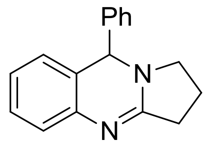

Compound 14: Following the general copper-oxidation procedure, **14** was obtained from aminal **13** after 24 h. **14** was isolated as a tan solid in 72% yield (0.1788 g). R_f 0.17 in EtOAc/MeOH/Et_3N 89:10:1 v/v/v); mp: 153–157 ºC; IR (KBr) 3061, 3023, 2919, 2859, 1620, 1590, 1567, 1479, 1454, 1432, 1299, 1285, 1196, 1031, 939, 878, 764, 737, 699, 602 cm^{-1}; ^1H NMR (500 MHz, $CDCl_3$) 7.43–7.25 (comp, 5H), 7.23–7.10 (comp, 2H), 6.91 (ddd, *J* = 8.2, 6.0, 2.6 Hz, 1H), 6.73 (app d, *J* = 7.6 Hz, 1H), 5.65 (s, 1H), 3.25–3.17 (m, 1H), 3.17–3.09 (m, 1H), 2.84–2.69 (comp, 2H), 2.10–1.88 (comp, 2H); ^{13}C NMR (125 MHz, $CDCl_3$) δ 161.4, 142.7, 142.3, 128.8, 128.3, 128.0, 127.5, 127.1, 124.1, 123.8, 123.2, 61.6, 49.2, 31.8, 18.9; ESIMS (*m/z*): 249.2 $[M + H]^+$.

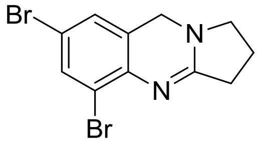

Compound 20: Following the general copper-oxidation procedure, using absolute ethanol instead of methanol, **20** was obtained from aminal **19** after 72 h. **20** was isolated as a tan solid in 18% yield (0.0578 g). R_f 0.25 in EtOAc/MeOH 90:10 v/v); mp: 164–167 ºC; IR (KBr) 2960, 2865, 1612, 1572, 1498, 1440, 1426, 1395, 1289, 1191, 1160, 855, 750, 734 cm^{-1}; ^1H NMR (500 MHz, $CDCl_3$) 7.53 (d, *J* = 2.2 Hz, 1H), 6.93 (d, *J* = 1.6 Hz, 1H), 4.51 (s, 2H), 3.33 (app t, *J* = 6.9 Hz, 2H), 2.72 (app t, *J* = 7.9 Hz, 2H), 2.04 (app p, *J* = 7.5 Hz, 2H); ^{13}C NMR (125

MHz, $CDCl_3$) δ 164.5, 141.1, 134.4, 127.9, 122.5, 119.9, 115.4, 51.4, 46.6, 31.7, 18.8; ESIMS (*m/z*): 331.1 $[M + H]^+$.

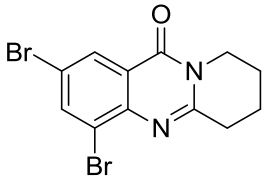

Compound 27: Following the general TBHP-oxidation procedure, **27** was obtained from aminal **26**. **27** was isolated as a tan solid in 50% yield (0.1775 g). R_f 0.21 in hexanes/EtOAc 80:20 v/v); mp: 171–173 ºC; IR (KBr) 3071, 2961, 1662, 1582, 1447, 1404, 1324, 1168, 1109, 993, 952, 878, 791, 754, 677 cm^{-1}; ^{1}H NMR (500 MHz, $CDCl_3$) 8.25 (d, J = 2.3 Hz, 1H), 8.02 (d, J = 2.3 Hz, 1H), 4.02 (app t, J = 6.1 Hz, 2H), 3.01 (app t, J = 6.6 Hz, 2H), 2.08–1.84 (comp, 4H); ^{13}C NMR (125 MHz, $CDCl_3$) δ 160.3, 156.3, 144.0, 139.9, 128.6, 122.4, 122.3, 118.7, 42.7, 32.1, 21.8, 19.0; ESIMS (*m/z*): 359.1 $[M + H]^+$.

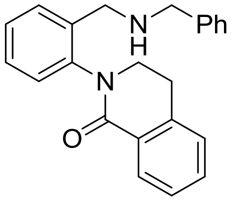

Compound 31: Following the general TBHP-oxidation procedure, **31** was obtained from aminal **30**. **31** was isolated as a tan oil in 48% yield (0.1626 g). R_f 0.29 in EtOAc/MeOH 98:2 v/v); IR (KBr) 3307, 3060, 3027, 2892, 2850, 2359, 2341, 1652, 1602, 1577, 1492, 1473, 1453, 1415, 1328, 1228, 1172, 1123, 1032, 742, 700 cm^{-1}; ^{1}H NMR (500 MHz, $CDCl_3$) 8.14 (app d, J = 7.7 Hz, 1H), 7.58 (dd, J = 5.7, 3.5 Hz, 1H), 7.49 (app td, J = 7.4, 1.4 Hz, 1H), 7.45–7.31 (comp, 3H), 7.31–7.19 (comp, 4H), 7.19–7.09 (comp, 3H), 3.94 (ddd, J = 12.2, 10.7, 4.7 Hz, 1H), 3.89–3.60 (comp, 5H), 3.10–2.88 (comp, 2H), 2.05 (br s, 1H); ^{13}C NMR (125 MHz, $CDCl_3$) δ 164.5, 141.9, 140.0, 138.5, 137.2, 131.9, 130.4, 129.4, 128.5, 128.4, 128.2, 128.1, 127.9, 127.0, 126.9, 126.8, 126.7, 53.6, 49.8, 49.1, 28.4; ESIMS (*m/z*): 343.5 $[M + H]^+$.

References

1) Sharpless, K. B.; Verhoeven, T. R. *Aldrichimica Acta*, **1979**, *12*, 63.
2) Zhang, C.; De, C. K.; Mal, R.; Seidel, D. *J. Am. Chem. Soc.*, **2008**, *130*, 416.
3) Richers, M. T.; Deb, I.; Platonova, A. Y.; Zhang, C.; Seidel, D. *Synthesis*, **2013**, *45*, in press.
4) Dieckmann, A.; Richers, M. T.; Platonova, A. Y.; Zhang, C.; Seidel, D.; Houk, K. N., *J. Org. Chem.*, **2013**, *78*, 4132.
5) Zhang, C.; Murarka, S.; Seidel, D. *J. Org. Chem.*, **2009**, *74*, 419.
6) Murai, K.; Komatsu, H.; Nagao, R.; Fujioka, H. *Org. Lett.* **2012**, *14*, 772.
7) Liu, J.-F.; Ye, P; Sprague, K.; Sargent, K.; Yohannes, D.; Baldino, C. M.; Wilson, C. J.; Ng, S.-C. *Org. Lett.* **2005**, *7*, 3363.
8) Decker, M. *Eur. J. Med. Chem.*, **2005**, *40*, 305.
9) Zhuo, C.; Hu, G.; Li, D.; Chen, J.; Li, Y.; Zhou, H.; Xie, Y. *Bioorg. Med. Chem.*, **2009**, *17*, 2351.
10) Gil, C.; Bräse, S. *Chem. Eur. J.*, **2005**, *11*, 2680.
11) Bowman, W. R.; Elsegood, M. R. J.; Stein, T.; Weaver, G. W. *Org. Biomol. Chem.*, **2007**, *5*, 103.
12) Ojo, B.; Chowdhury, B. K. *Synth. Comm.*, **2012**, *42*, 1002.
13) Zhang, C.; De, C. K.; Seidel, D. *Org. Synth.*, **2012**, *89*, 274.

^1H NMR of **8** in $CDCl_3$

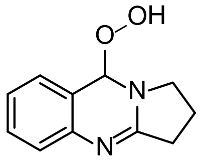

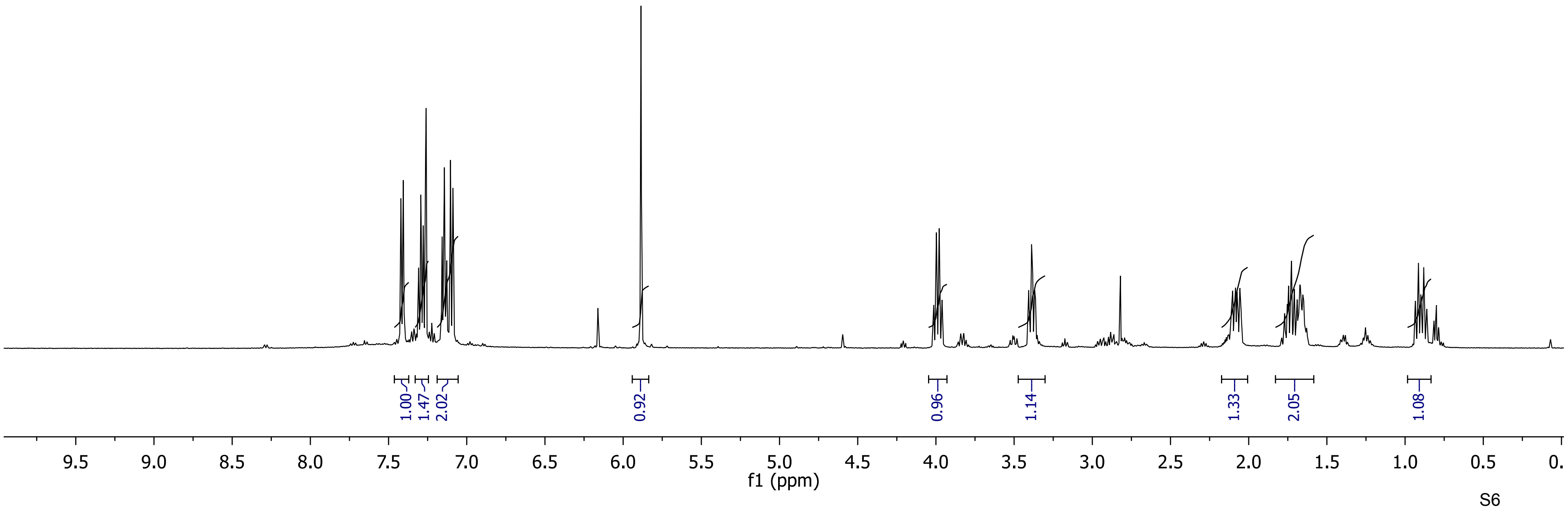

^{13}C NMR of **8** in $CDCl_3$

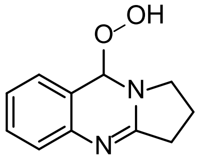

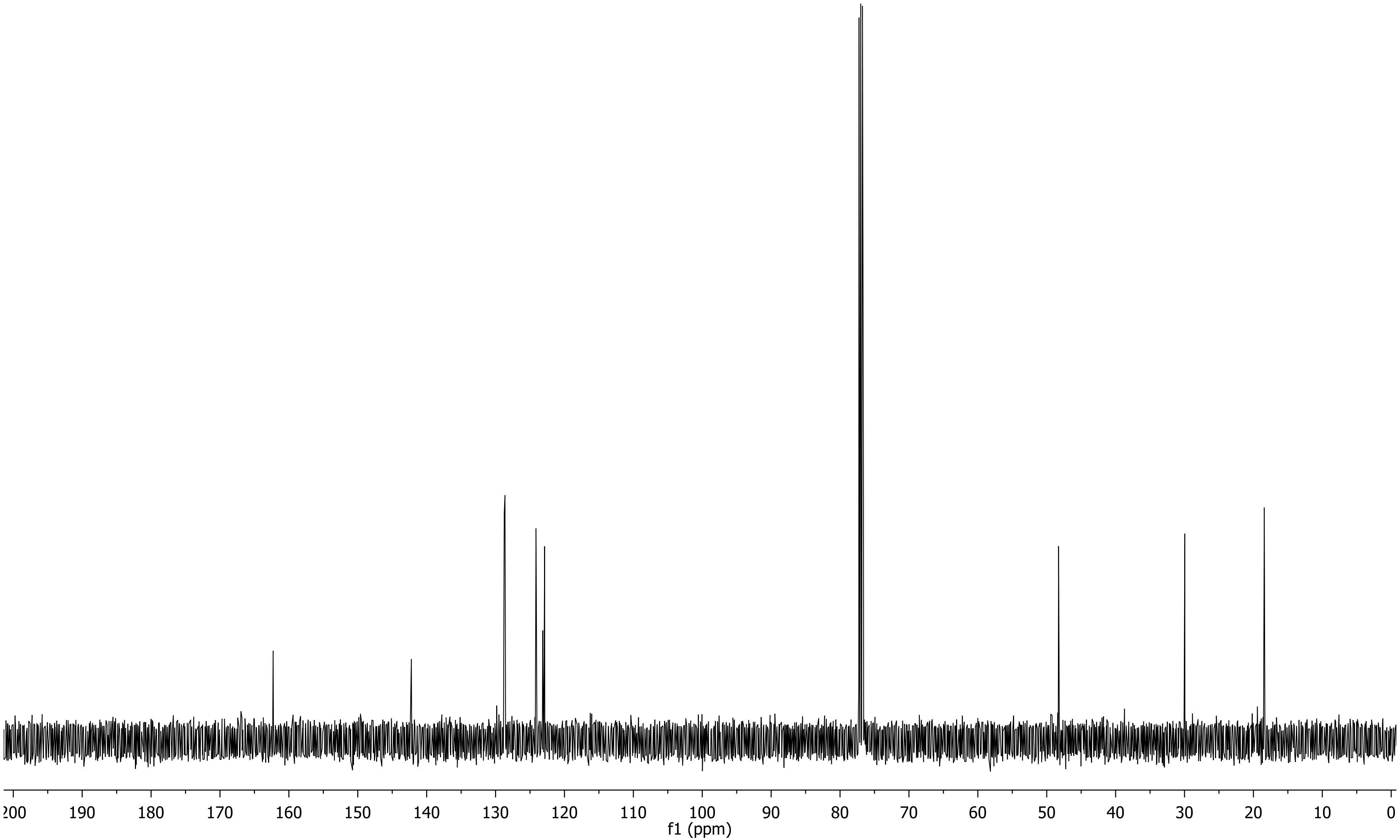

^{1}H NMR of **10** in $CDCl_3$

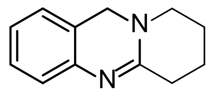

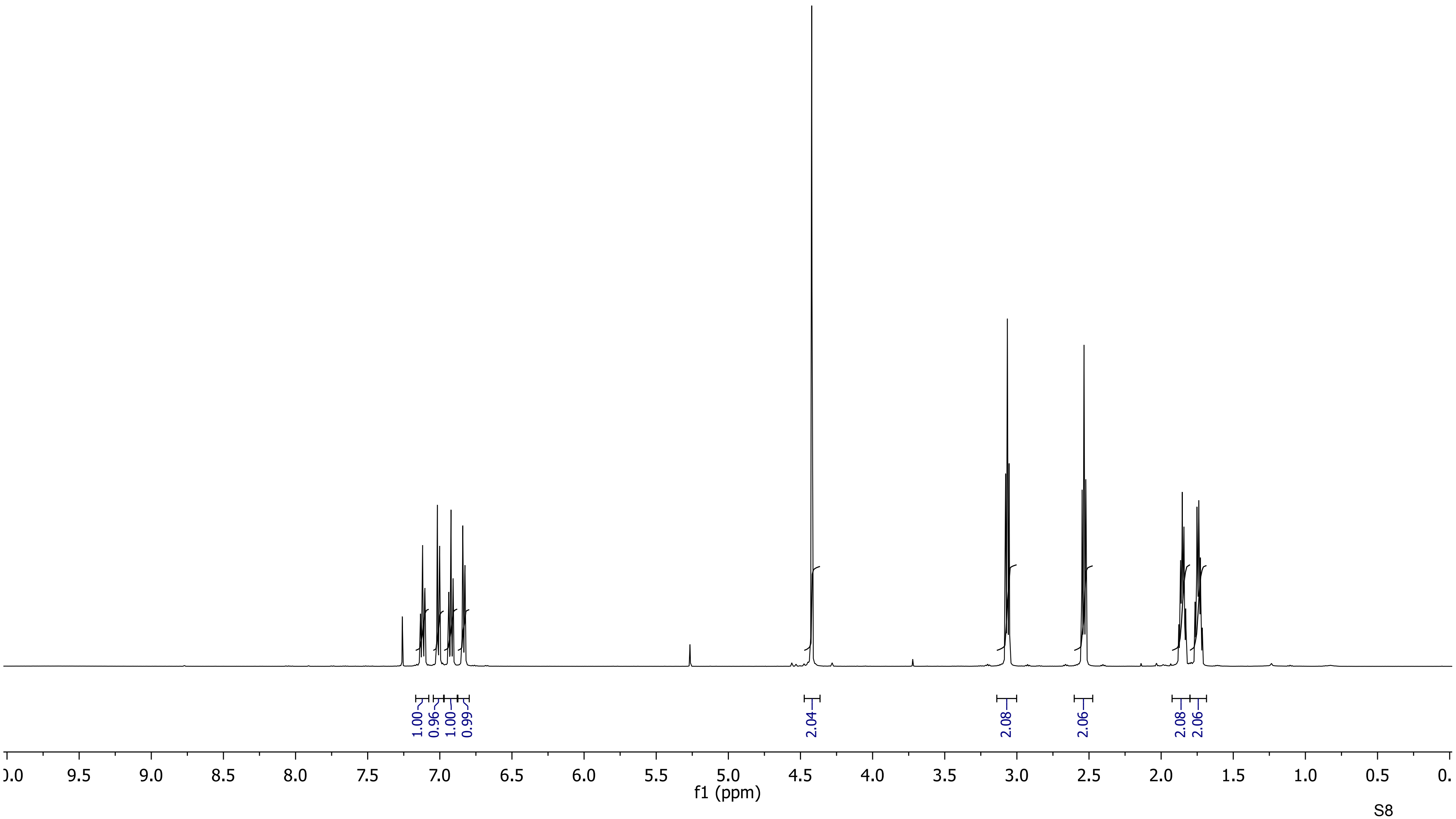

^{13}C NMR of **10** in $CDCl_3$

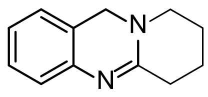

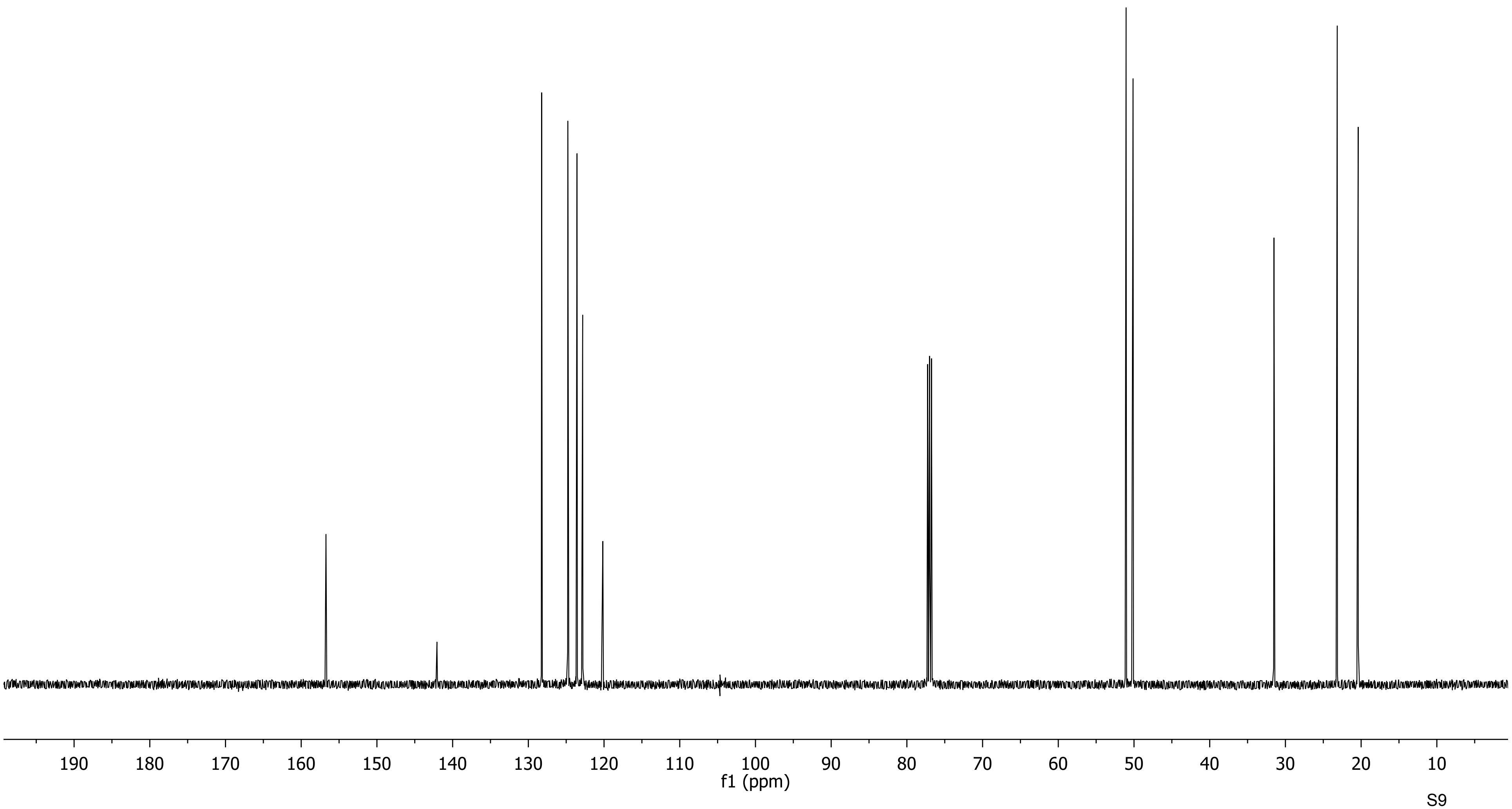

^{1}H NMR of **11** in $CDCl_3$

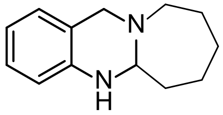

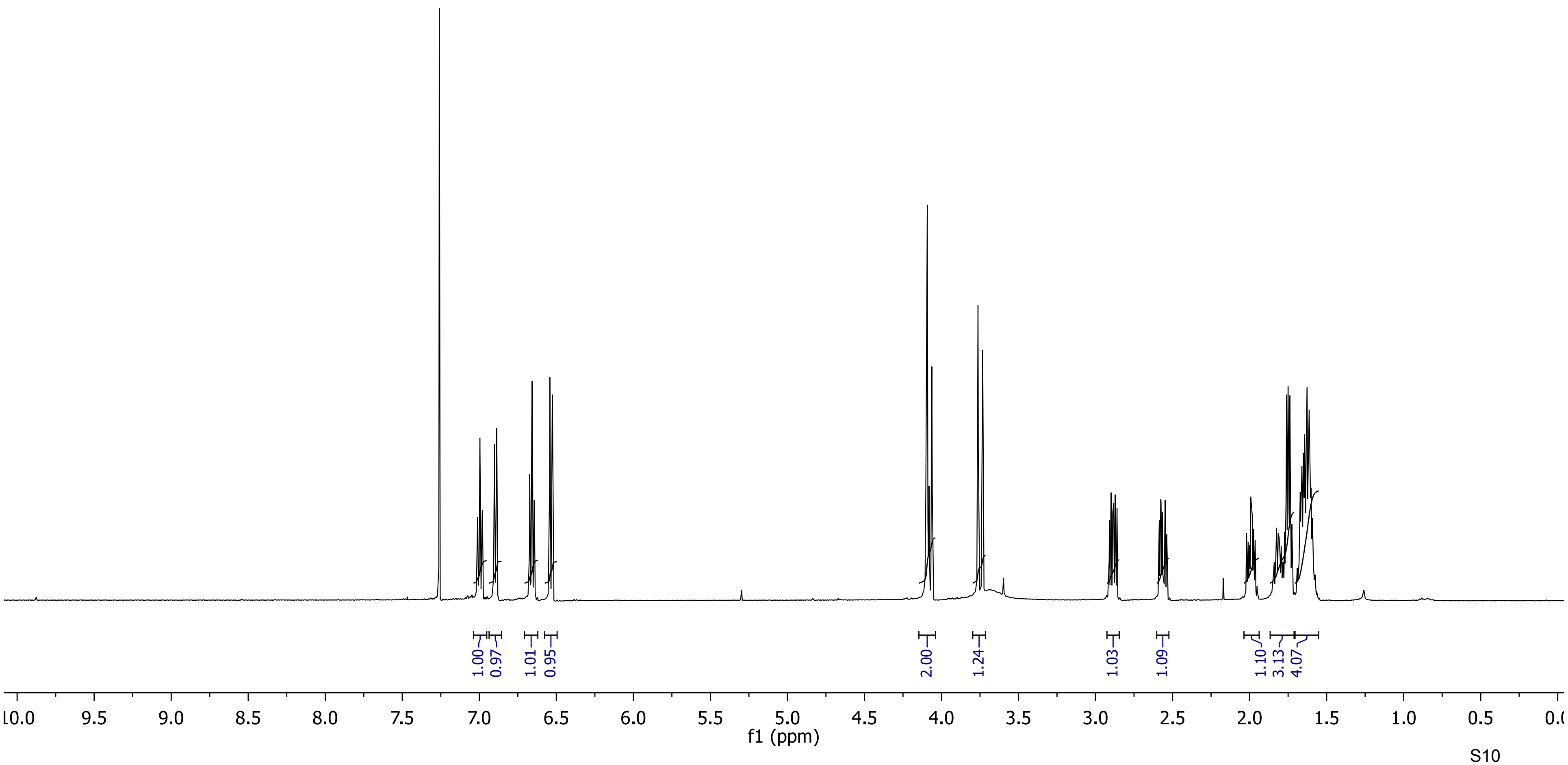

^{13}C NMR of **11** in $CDCl_3$

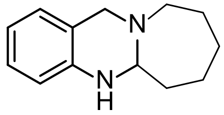

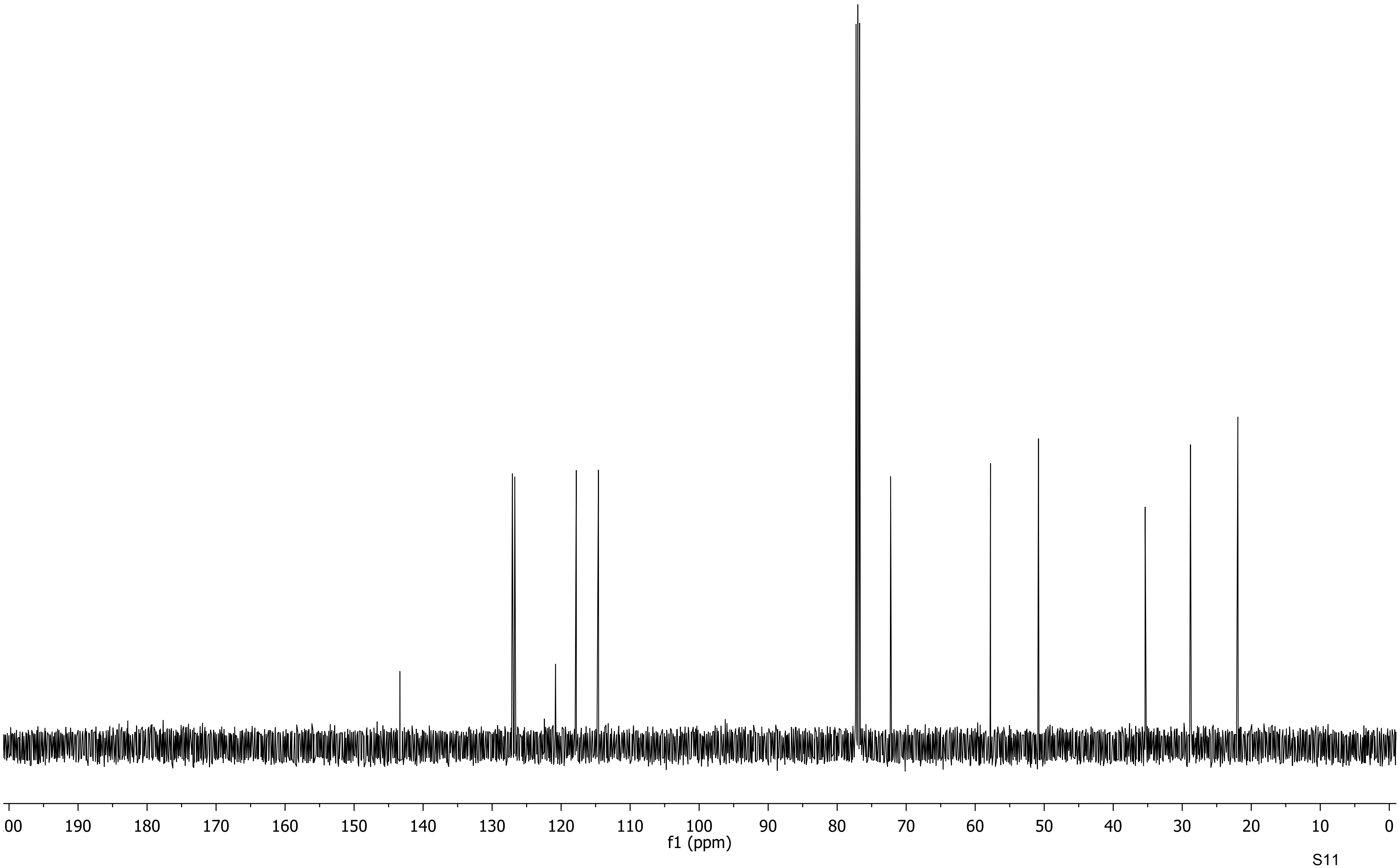

^1H NMR of **12** in $CDCl_3$

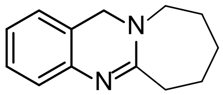

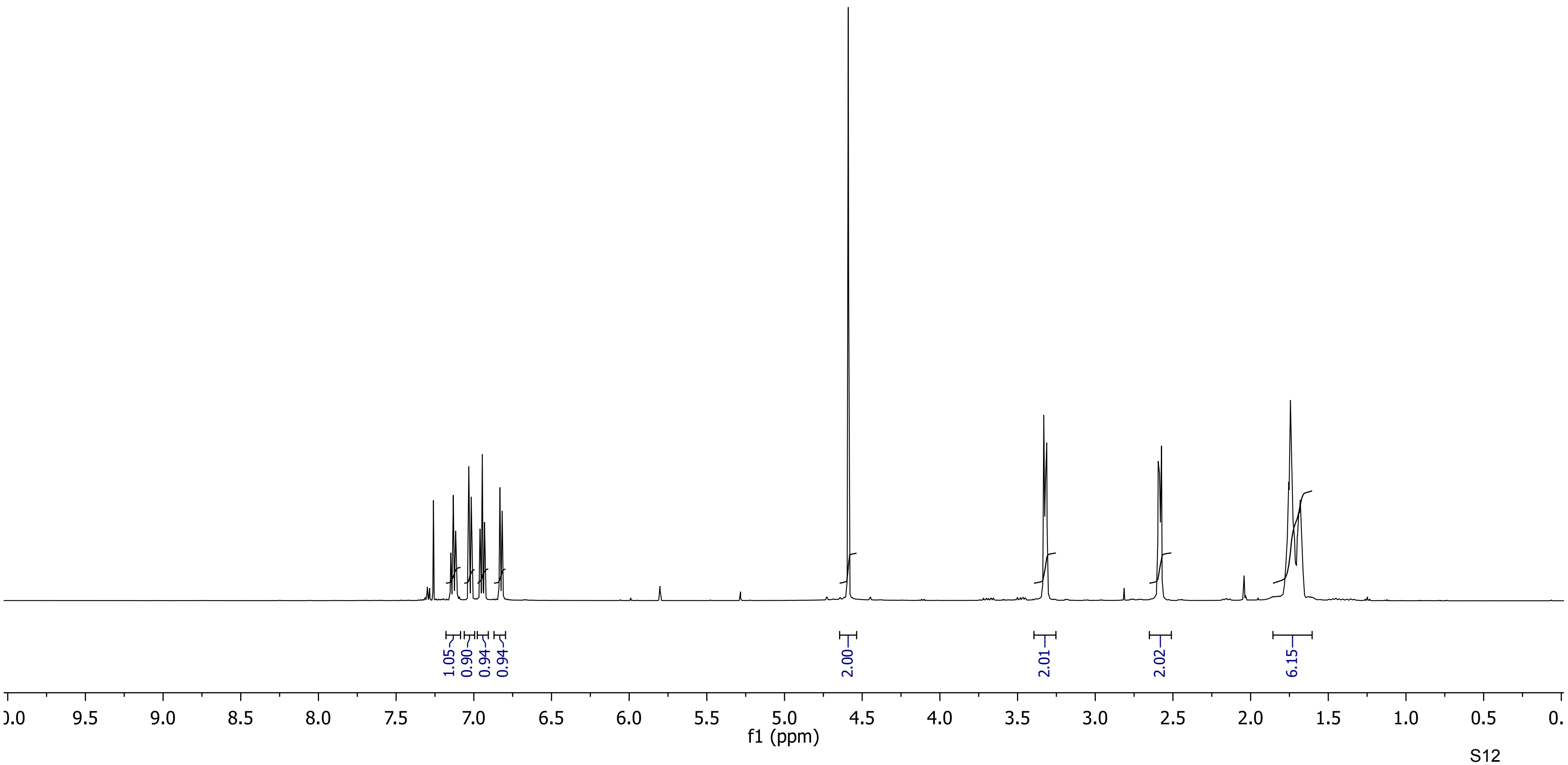

^{13}C NMR of **12** in $CDCl_3$

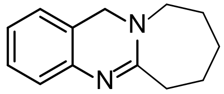

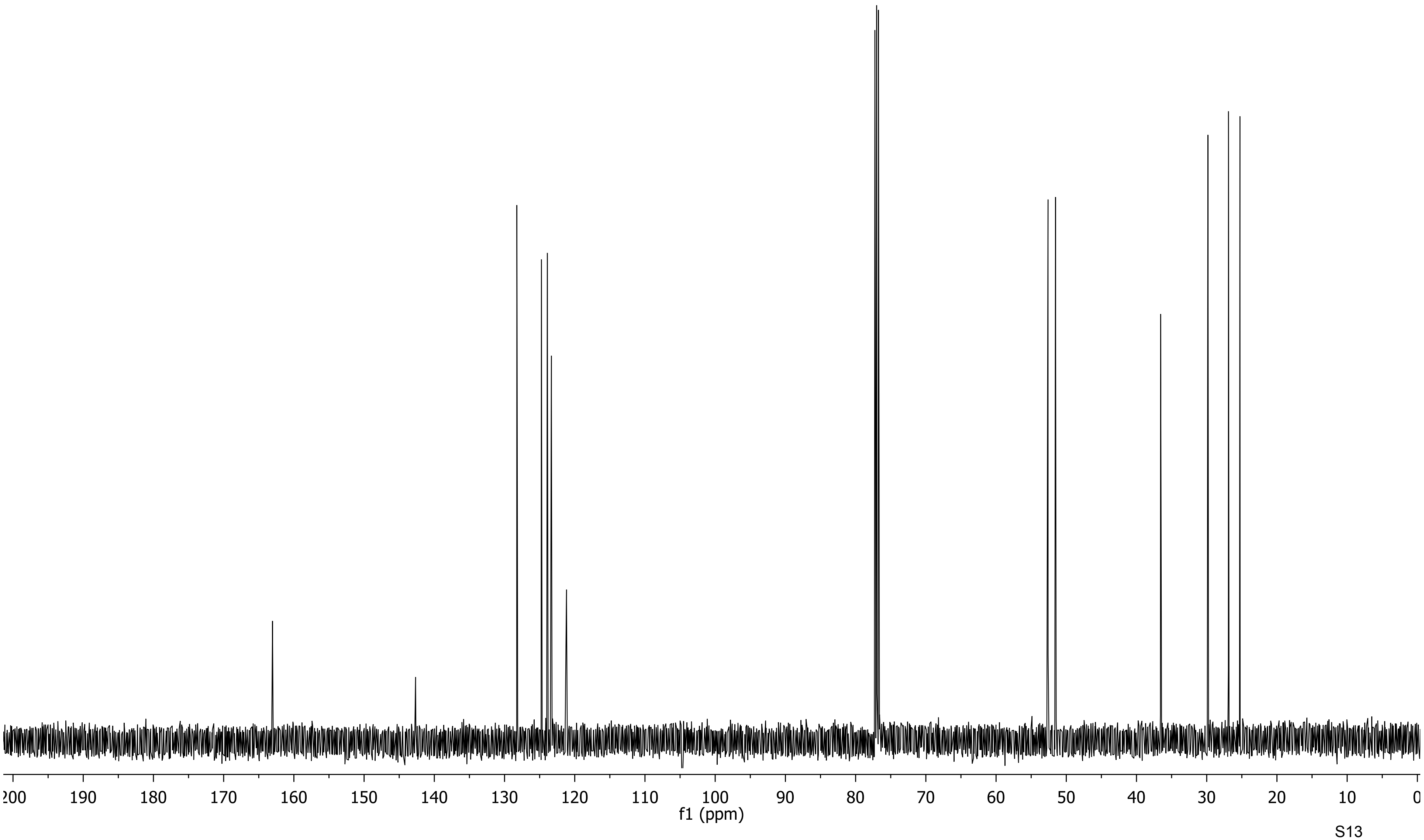

^1H NMR of **14** in $CDCl_3$

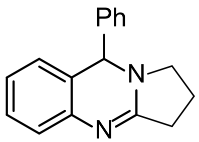

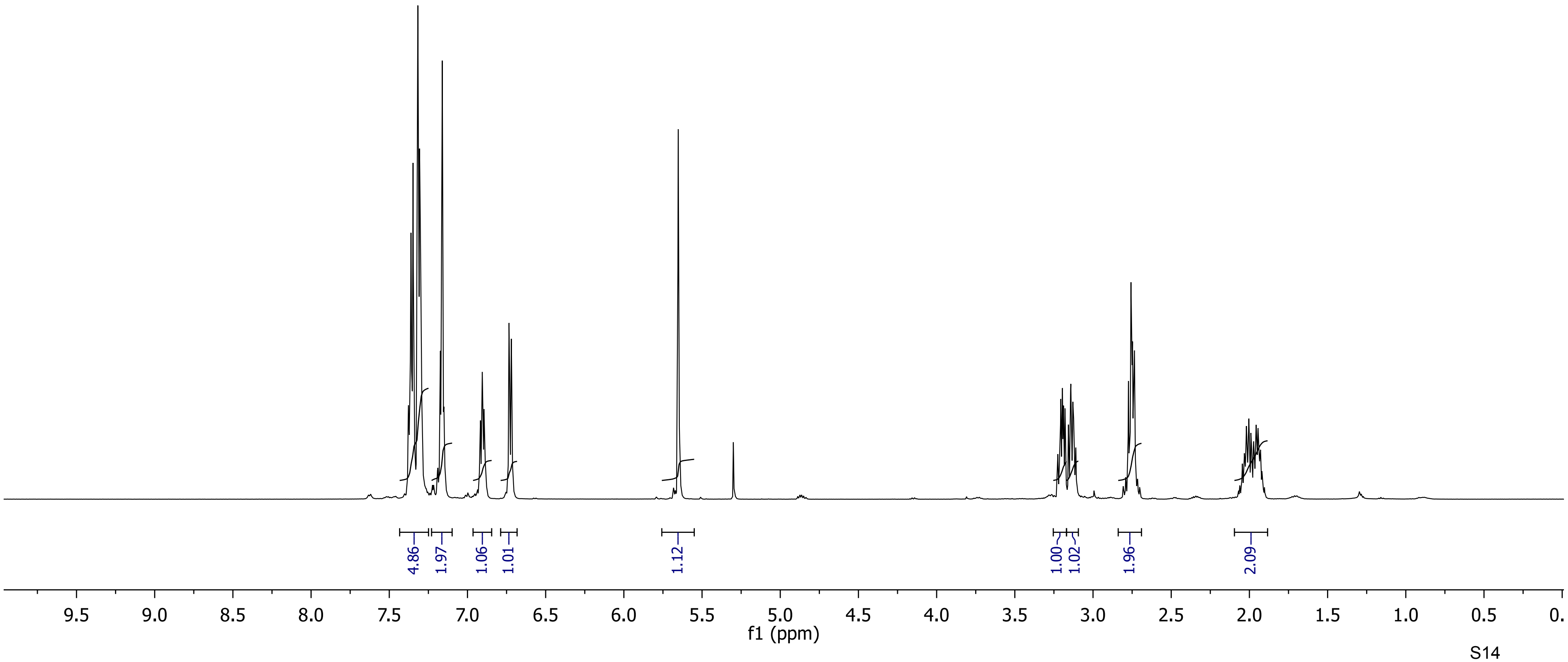

^{13}C NMR of **14** in $CDCl_3$

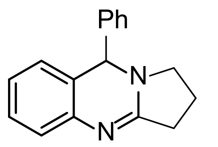

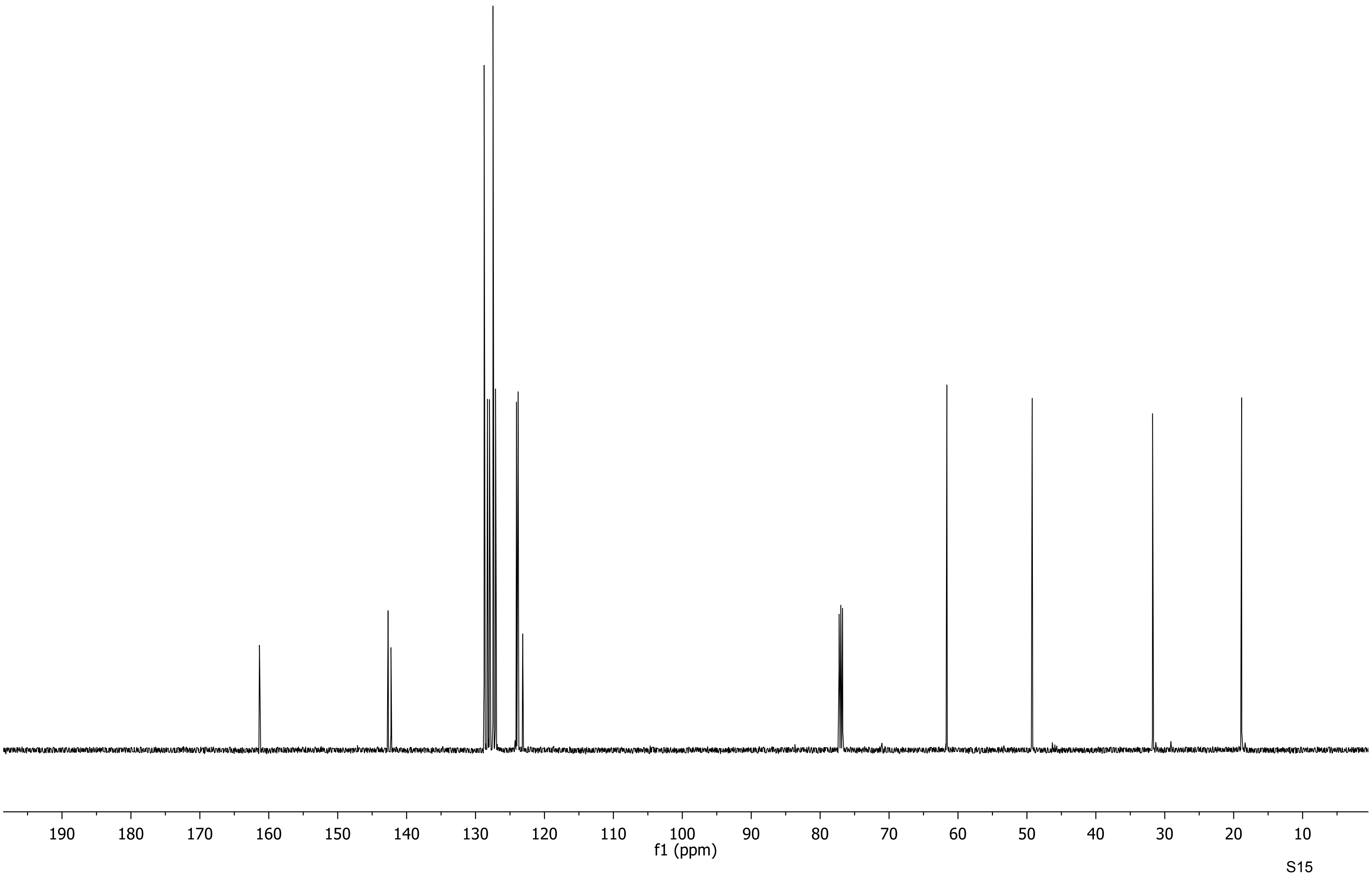

^1H NMR of **20** in $CDCl_3$

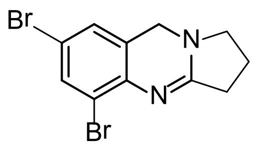

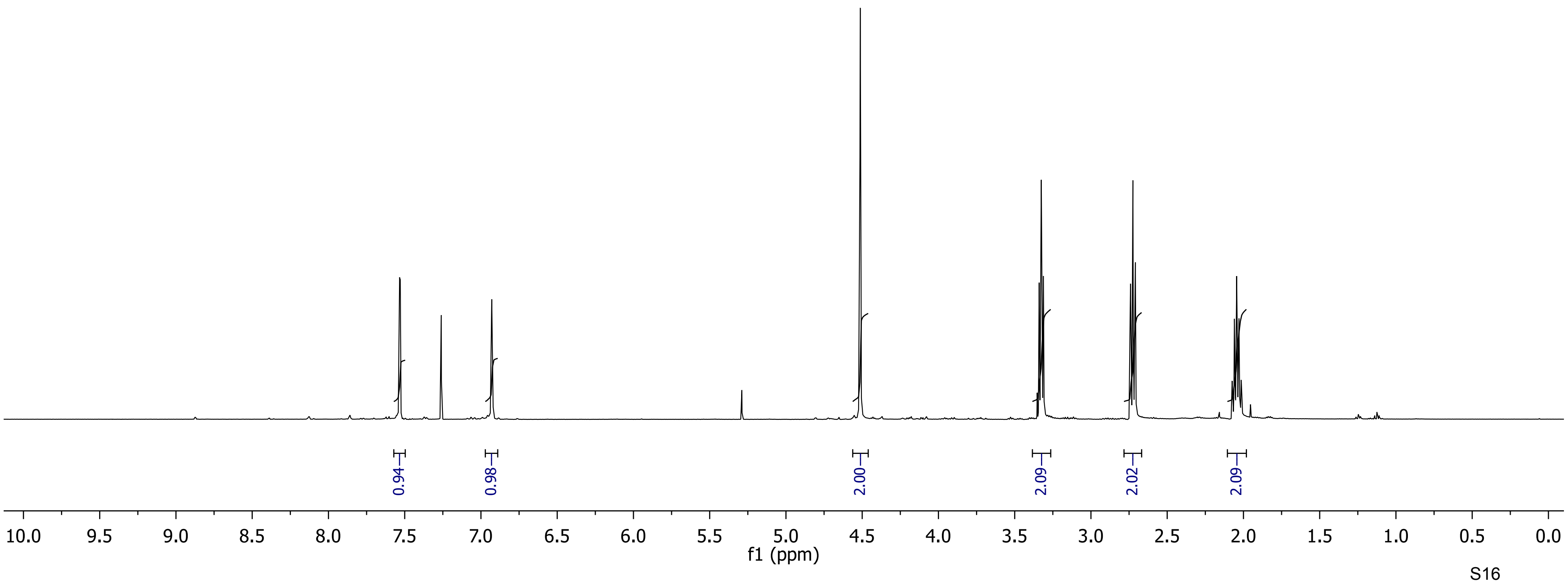

^{13}C NMR of **20** in $CDCl_3$

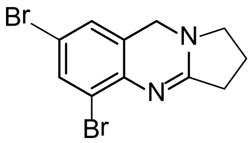

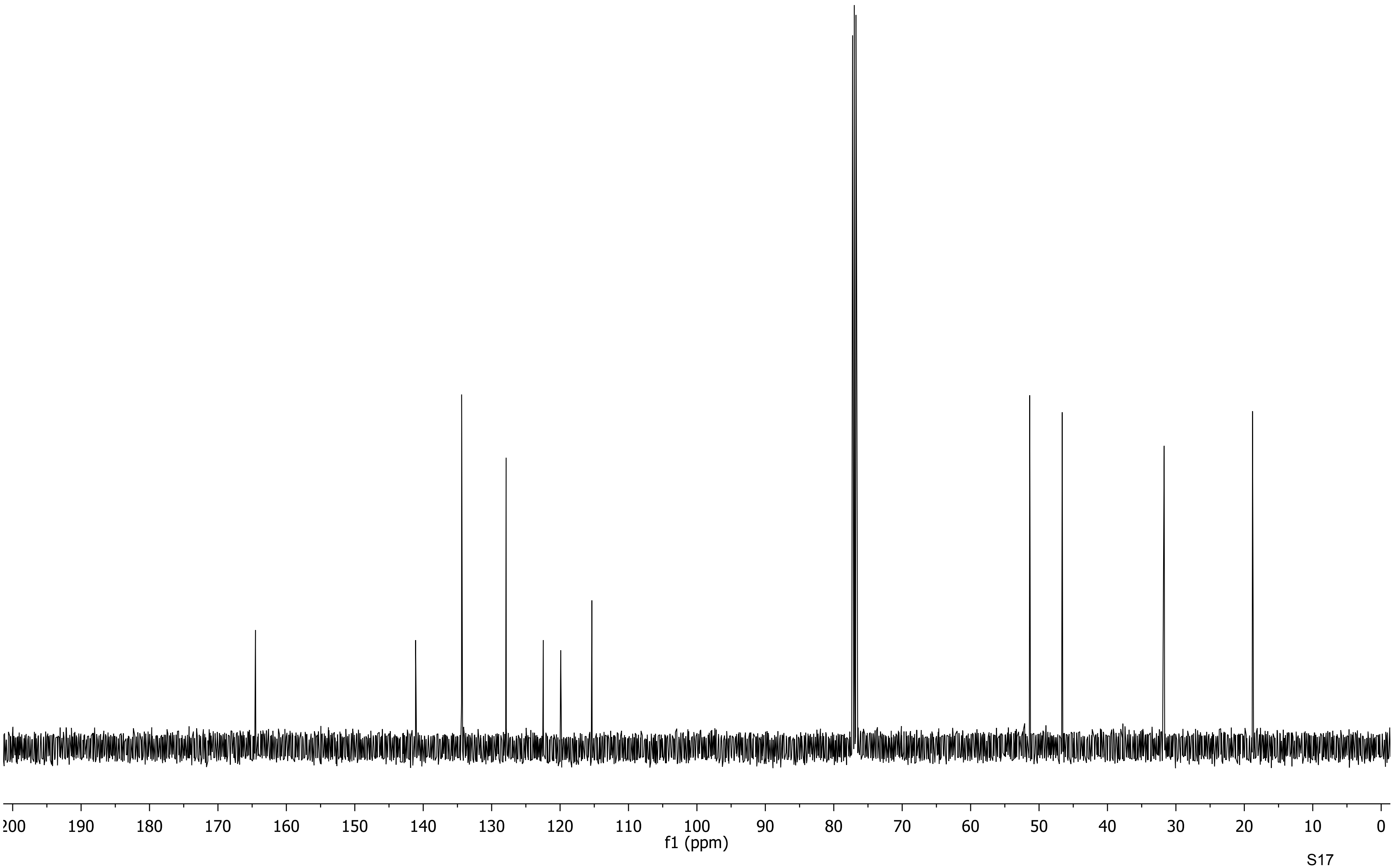

^1H NMR of **27** in $CDCl_3$

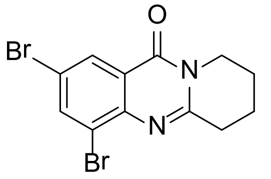

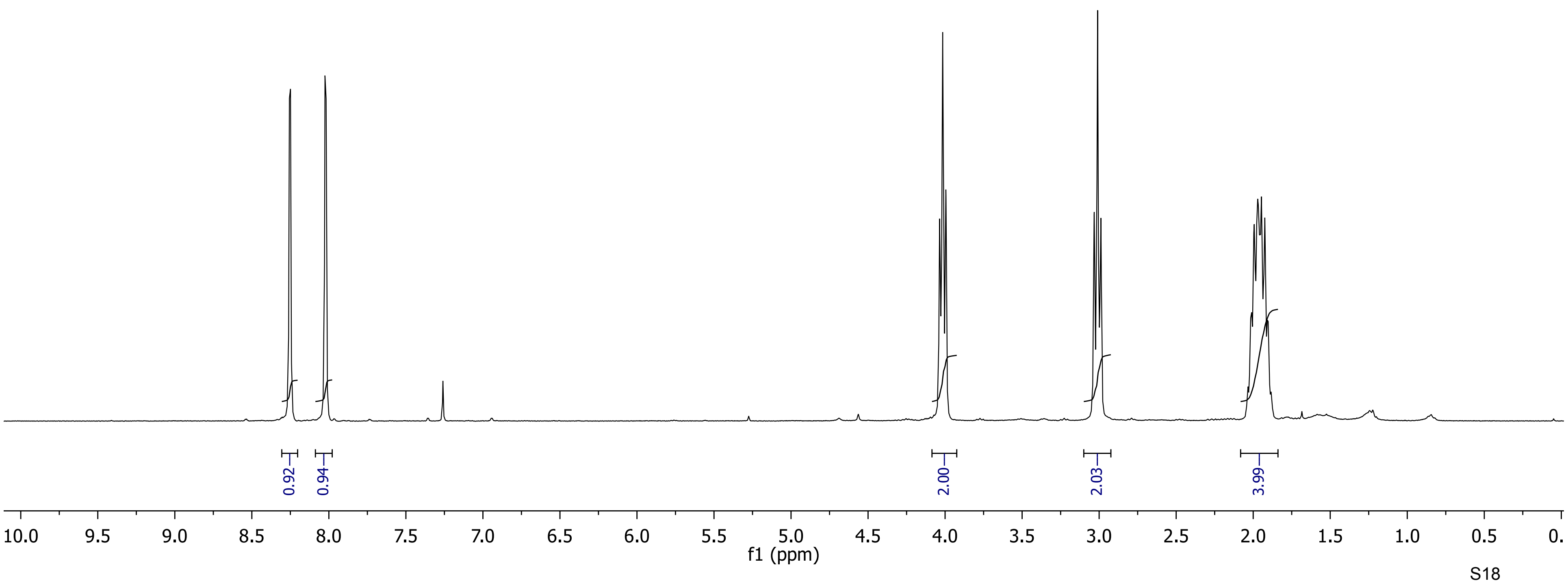

^{13}C NMR of **27** in $CDCl_3$

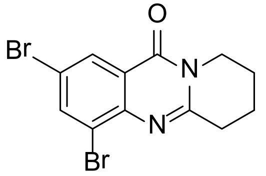

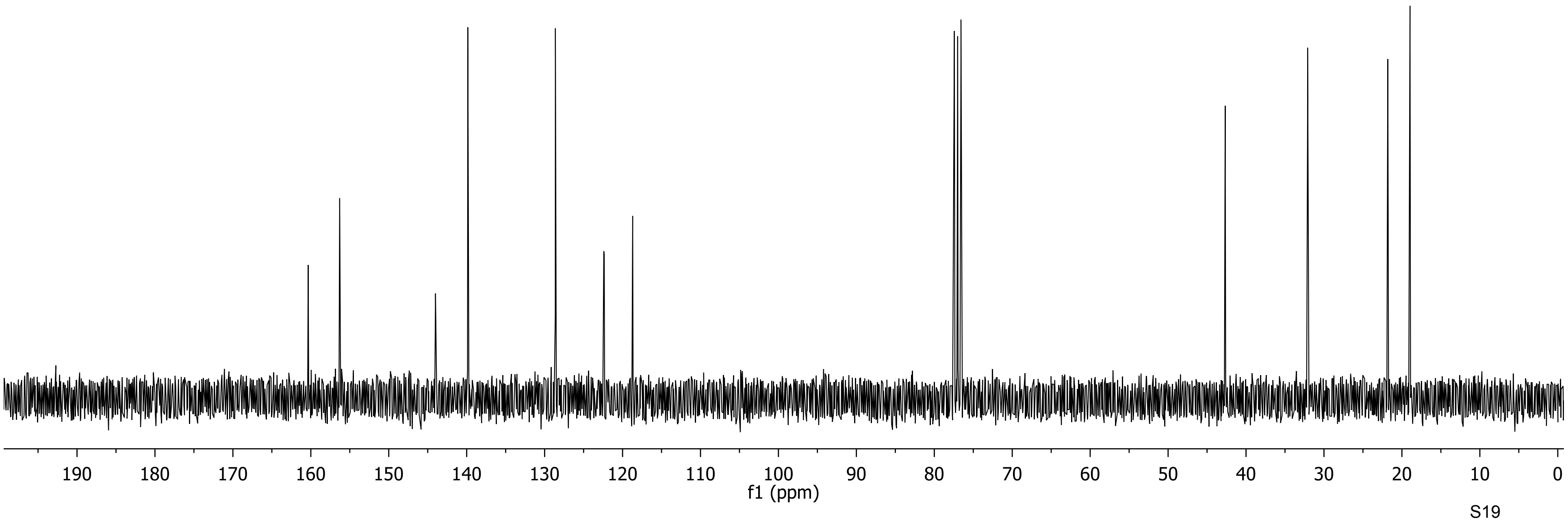

^{1}H NMR of **31** in $CDCl_3$

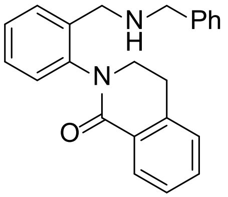

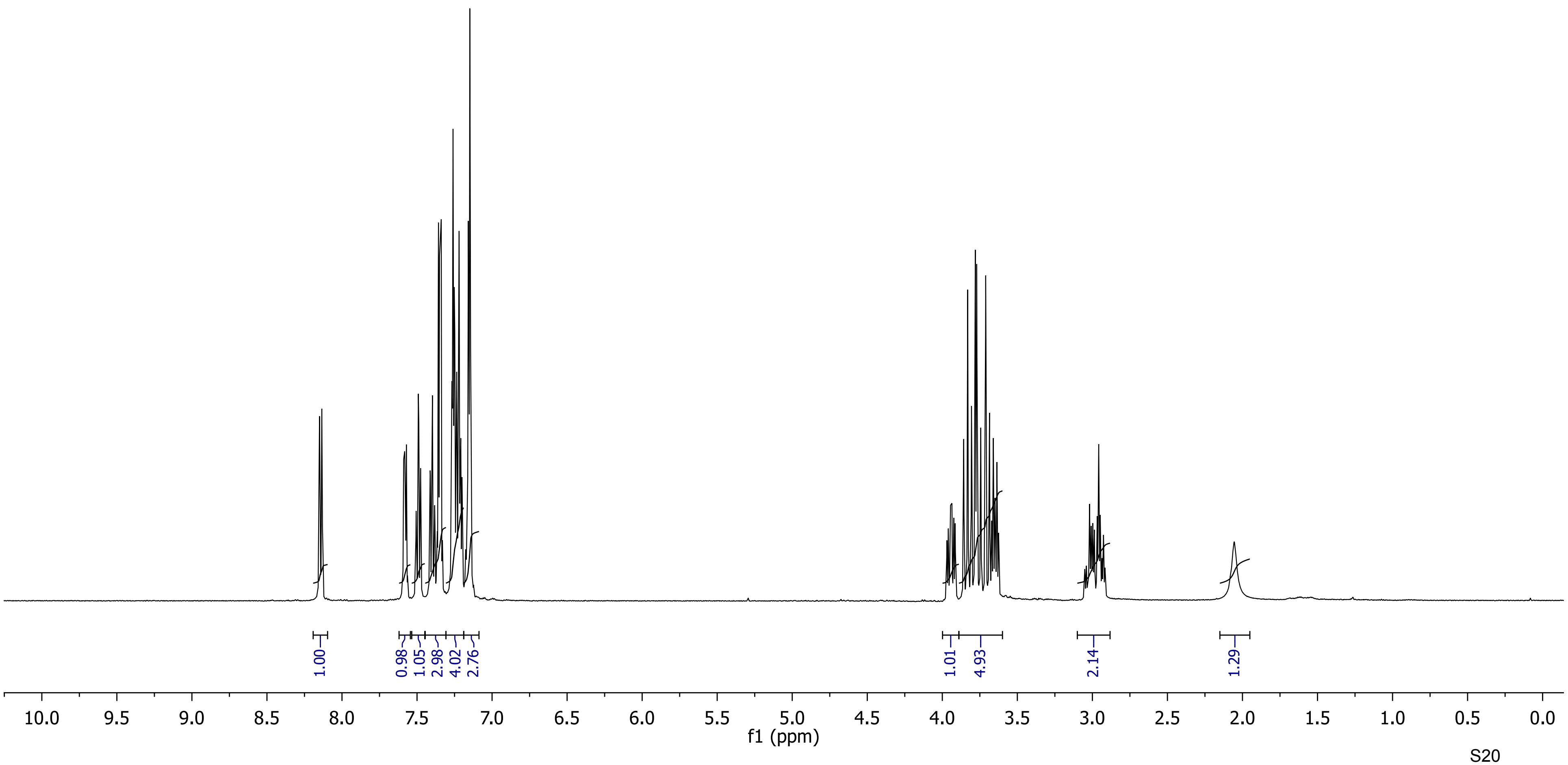

^{13}C NMR of **31** in $CDCl_3$

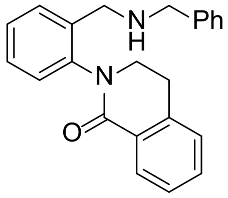

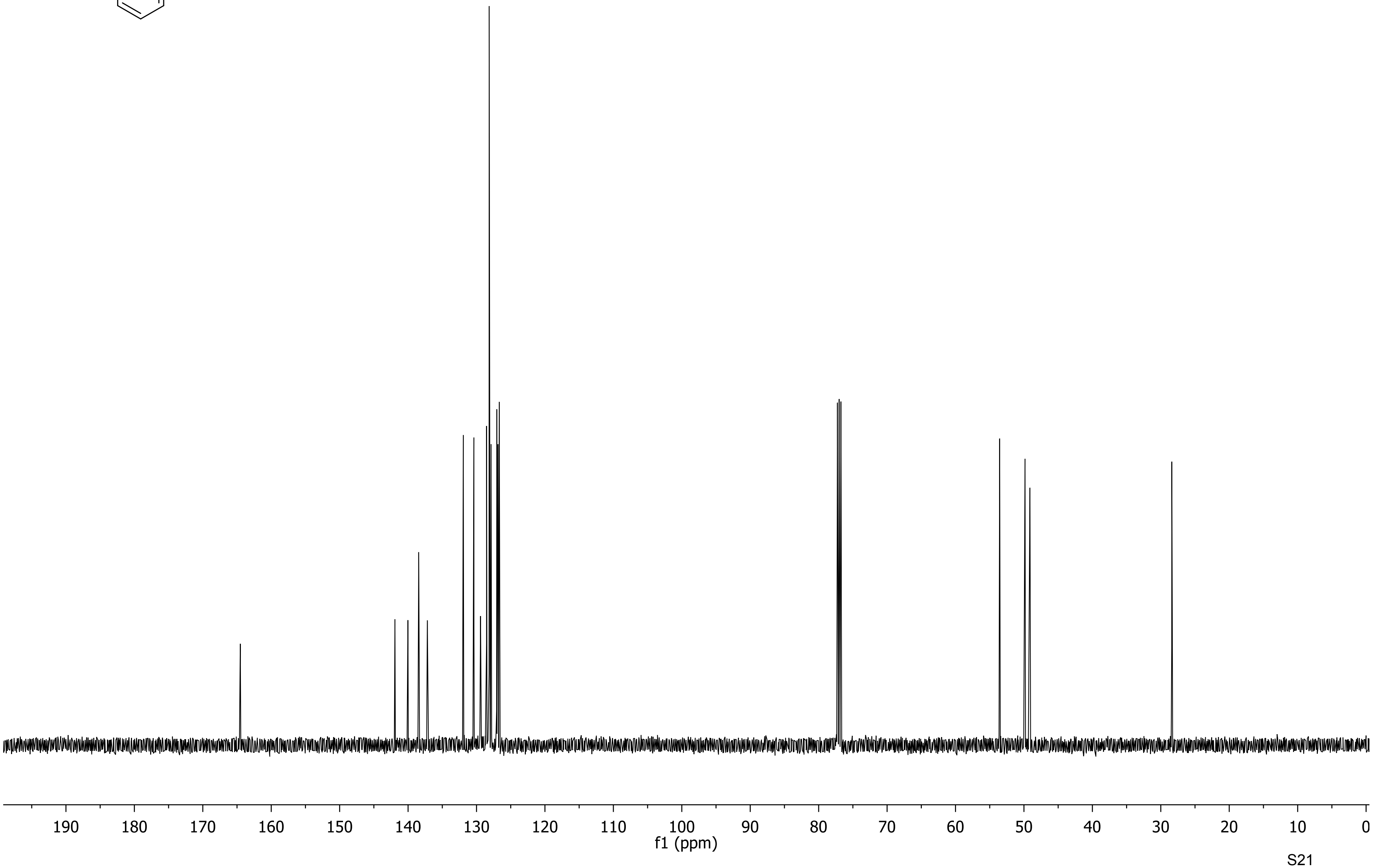

Supplement: File 1 — Experimental details, characterization data and 1H and 13C NMR spectra for all new compounds. [file Beilstein_J_Org_Chem-09-1194-s001.pdf]
